# Enhanced Charge Transfer Kinetics in Hematite Photoanodes via Au Nanoclusters—Toward Efficient Light-Driven Hydrogen Generation

**DOI:** 10.1021/acs.langmuir.4c04843

**Published:** 2025-05-01

**Authors:** Aleksandra Szkudlarek, Kamila Kollbek, Krzysztof Mech, Krzysztof Maćkosz, Mateusz Marzec, Vitaliy Bilovol, Marcin Sikora

**Affiliations:** 1 Academic Centre for Materials and Nanotechnology, AGH University of Krakow, av. Mickiewicza 30, Krakow 30-059, Poland; 2 Department of Solid State Physics, Faculty of Physics and Applied Computer Science, AGH University of Krakow, av. Mickiewicza 30, Krakow 30-059, Poland; 3 Advanced Materials and Surfaces, Mechanics of Materials and Nanostructures, EMPA, Swiss Federal Laboratories for Materials Science and Technology, Feuerwerkerstrasse 39, Thun 3602, Switzerland; 4 National Synchrotron Radiation Centre SOLARIS, Jagiellonian University, Czerwone Maki 98, Krakow 30-392, Poland

## Abstract

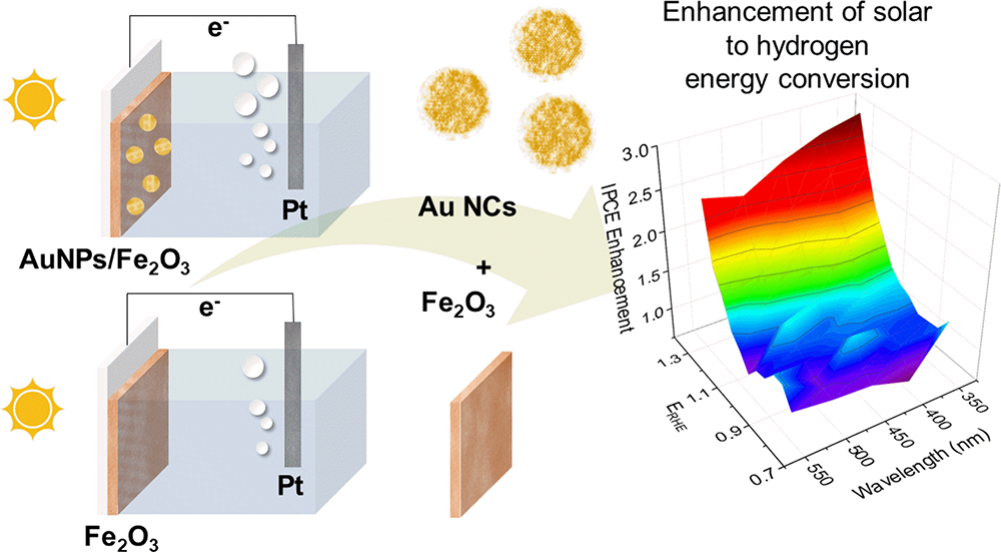

In these studies, we explain the mechanism by which gold nanoparticles enhance the photoelectrochemical activity of thin films of hematite. The electrodes were manufactured using magnetron sputtering in combination with inert gas condensation, one of the most advanced techniques to decorate the top material layer with metallic nanoclusters. Adjusting the very low surface concentration of widely separated Au nanoclusters, corresponding to 2.3% surface coverage, we observed a 2-fold increase in photocurrent values. This effect can be explained by electrochemical impedance spectroscopy results, which show an increase in charge carriers density by 1.7 and charge carriers lifetime by 1.4. Comprehensive in-depth microscopic and spectroscopic studies of the morphological, chemical, and electronic properties of hematite allow not only the characterization of the material but also the determination of the role of metallic Au nanoclusters at the electrode–electrolyte interface. Understanding the mechanism of the interactions between hematite and metallic Au nanoclusters is a key factor in designing advanced sustainable devices for solar-to-chemical energy conversion.

## Introduction

Iron oxides have been known for centuries to have interesting magnetic, catalytic, and optical properties and exist in many different forms with the most popular complexes, known as wüstite (FeO), magnetite (Fe_3_O_4_), maghemite (γ-Fe_2_O_3_), and hematite (α-Fe_2_O_3_). Hematite is an antiferromagnetic material that naturally occurs as a mineral. The peculiar photoelectrochemical properties (electronic bandgap in the visible range, robustness to work in alkaline and base solutions, and high adsorption capacity) make the material very interesting for constructing anodes designed for photoelectrochemical water-splitting. This, together with high long-term stability, abundance in nature, nontoxicity, and low price, gives rise to a wide potential range of applications in cost-effective solar energy conversion systems.^1−5^

Although this set of physicochemical properties is unique, the material is not widely used in light-harvesting devices due to its low solar energy conversion factor, which arises from the poor conductivity and short lifetime of the photogenerated charge carriers before recombination, implicating small diffusion length and misalignment of the conduction band with respect to the potential level of hydrogen reduction.^6^ Furthermore, photoelectrochemical applications are restricted by the high value of the onset potential and the low Faradaic oxidation rate.^7,8^ To overcome these issues, several approaches have been proposed, primarily based on nanostructured hematite forms^6^ with TiO_2_^9^ or SnO_2_ scaffolds,^10^ doping with Ti and Si,^11^ Zr or Sn,^12^ and also by introducing intermediate layers as electron collectors such as TiO_2_^9^ or passivation overlayers such as Ta_2_O_5_.^13^ Although these strategies have been successful to some extent, the fundamental limit of the material lies in the fact that only a part of the absorbed photons result in mobile charge carriers, whereas a significant fraction leads to localized states, which do not contribute to the photocurrent.^14^

Our studies investigated the role of gold nanoclusters (AuNCs) deposited on the top of the hematite photoanode. Modification of the hematite surface with Au nanoclusters was previously investigated by Gross Koren et al.,^15^ Li et al.,^16^ and Tofanello^17^ in an attempt to distinguish the contributions originating from the plasmonic effect, formation of the Schottky junction at the interface, or enhanced catalytical activity. The primary focus of the authors was directed toward the impact of localized surface plasmon resonance stemming from the incorporation of Au nanoclusters on the enhancement of the light-harvesting efficiency in photoelectrochemical water splitting. In our work, throughout the series of preliminary samples, we optimized the method of the fabrication of hematite gold-decorated photoanodes by applying high-vacuum techniques using magnetron sputtering with inert gas condensation to preserve the optical characteristics. We adjusted the amount of Au nanoclusters to such a low concentration that the plasmon surface resonance effect was not observed.

The material morphology and chemical phase composition were widely characterized using several microscopic and spectroscopic techniques, such as scanning electron microscopy (SEM), transmission electron microscopy (TEM), and high-angle annular dark-field scanning transmission electron microscopy (HAADF-STEM). Chemical and phase compositions were investigated with energy dispersive X-ray spectroscopy (EDX) and Mössbauer spectroscopy, respectively. The electronic structure was studied by soft X-ray absorption spectroscopy (XAS). The optical properties of the deposited thin films were investigated by using UV–Vis spectroscopy. Work function was measured by ultraviolet photoelectron spectroscopy (UPS). The photocurrent maps were recorded as functions of applied potential and wavelength. The charge carriers’ density and lifetime were determined by electrochemical impedance spectroscopy (EIS).

## Experimental Section

α-Fe_2_O_3_ thin films were deposited using radio frequency magnetron sputtering (RF), and Au nanoclusters were deposited by inert gas condensation (IGC) using the commercial system by Mantis Deposition Ltd. Details about the IGC setup can be found in the work by Kusior.^18^ Since this process occurs under ultrahigh vacuum (UHV) conditions, it prevents contamination of the hematite film’s top layer. The commercial ITO substrates from Sigma-Aldrich were cleaned using a standard protocol: ultrasonic treatment in acetone for 5 min, followed by rinsing with isopropanol, an additional 5 min ultrasonic treatment in isopropanol, and finally, O_2_ plasma cleaning for 10 min. Anodes were prepared in two steps. First, a thin film of α-Fe_2_O_3_ was deposited on the ITO substrates. In the subsequent step, the surface was decorated with Au nanoclusters. Thin films were deposited using an RF magnetron source. The target surface was cleaned by sputtering for 12 min before the film deposition. Sputtering was performed in a controllable gas atmosphere containing Ar (flow rate 50 sccm). The pressure during the process was about 9.0 × 10^–3^ Torr, whereas the base pressure was about 5.7 × 10^–8^ Torr. The input power applied to the RF magnetron source was 130 W. The deposition rate was monitored by a quartz crystal microbalance. During the deposition procedure, the substrates were rotated at 20 rpm to ensure thin film uniformity. Au nanoclusters were deposited onto the α-Fe_2_O_3_ layer surface from a pure gold target (Kurt J. Lesker Company, purity: 99,99%). The magnetron source operated in direct current (DC) mode, with an input current of 100 mA. AuNCs deposition was performed at a constant pressure of 7.5 × 10^–4^ Torr, with argon (100 sccm) and helium (20 sccm) flow rates maintained throughout the 20 min process. Nanoclusters size was monitored in situ by a quadrupole mass spectrometer aligned with the cluster source. During the deposition process, the substrates were rotated at 20 rpm. All samples were deposited at room temperature and postannealed in an O_2_ atmosphere at 350 °C for 6 h. AFM scans were performed in air using a Bruker Dimension ICON XR microscope working in peak force tapping mode on both types of substrates, Si and ITO, with the following scan size of 1 μm and 256 lines and 0.601 Hz scan rate. The peak force tapping control parameters were adjusted as follows: the peak force amplitude to 140 nm, peak force frequency to 2 kHz, and lift height to 25 nm. The feedback gain and the peak force set point were set to 1.91 and 0.021 V, respectively. Scanning electron microscopy analyses were performed using a VERSA 3D Dual Beam System by FEI in analytical mode with a beam current of 4 nA, a beam energy of 30 keV, and a working distance of 1.5 mm. EDX was performed using an Ultim MAX 40 spectrometer from Oxford Instruments at 5 keV beam energy and 4 nA beam current. HAADF-STEM images of the thin film of α-Fe_2_O_3_ were taken using probe-corrected Thermo Fisher Scientific Titan Themis 200G. The TEM lamella was prepared using the standard lift-out method with a Tescan Lyra dual FIB-SEM system. The distribution and crystal phase of Au nanoclusters were investigated using an HR TEM Tecnai TF 20 X-TWIN by FEI with a beam energy of 200 keV. The nanoclusters were deposited on an ultrathin holey carbon support film during the sputtering process. XAS measurements were performed at the PIRX beamline at the Solaris National Synchrotron Radiation Centre, Poland.^19^ Spectra were collected at the K edge of oxygen and the L_2,3_ edge of iron using total electron yield (TEY) detection probed via sample neutralization current without bias voltage. Measurements were performed at room temperature under ultrahigh vacuum (*P* < 1 × 10^–9^ mbar) on ex situ loaded films.

The optical response–absorbance/transmittance was measured using a UV–Vis–NIR Lambda 750 spectrophotometer from PerkinElmer without an integration sphere. The absorption of hematite was calculated by subtracting the absorption curve of the ITO substrate. EIS measurements were performed using a Biologic SP-200 potentiostat/galvanostat (Bio-Logic Science Instruments) equipped with the EIS module. The measurements were carried out using the standard three-electrode setup (Pt gauze as the counter electrode and Ag/AgCl leakless electrode as the reference electrode). The synthesized materials were deposited onto the surface of the ITO glass substrates and then applied as working electrodes. The electrochemically active surface area of the electrodes amounted to 0.5 cm^2^. EIS spectra were recorded at 0.22 V (vs Ag/AgCl) in 1 M NaOH solution purged for 15 min with Ar. Measurements were performed at frequencies ranging from 4 MHz to 50 Hz and a potential amplitude of 5 mV under dark conditions.

Mott–Schottky measurements were recorded at a frequency of 1 kHz in a potential range of 0.1 to −0.6 V (vs Ag/AgCl). EIS spectra were fitted and analyzed by using EC-Lab software.

All potentials were recalculated and presented versus the RHE electrode (eq 1).
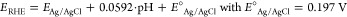
1

The photocurrent spectra were collected using an apparatus equipped with a 150 W xenon lamp, a monochromator, a shutter, and a precise sensitive potentiostat. The setup was built by Insytytut Fotonowy (Krakow, Poland). The sample was mounted in a photoelectrochemical cell using Pt wire as a counter electrode and Ag/AgCl (3.5 M KCl) as a reference electrode. The illuminated circular area was 1 cm in diameter. The spectra were recorded in 1 M NaOH. The direction of light irradiation was perpendicular to the surface from the backside of the film.

Light intensity was measured using an S120VC high-sensitive photodiode by Thorlabs, with an active aperture of 9.5 mm in diameter.

UPS measurements were performed in a PHI VersaProbeII apparatus (ULVAC-PHI, Chigasaki, Japan) using the He I line (21.22 eV) from a UHV gas discharge lamp. The acceleration potential of −5 V was applied to the sample, leading to a much more pronounced secondary electron cutoff (SE cutoff). The work function (measured as the difference between photon energy and SE cutoff position) and the hole injection barrier (given by the difference between the substrate Fermi level and the HOMO onset of the material) were determined. For each UPS spectrum, the emission features originating from the secondary line excitations of the He I gas discharge were subtracted. The measurement times were kept as short as possible to avoid any possible degradation of the examined materials during exposure to UV radiation. The measurements were done before and after the argon gas cluster ion beam sputtering of samples. The sputtering process was carried out with approximately 4000 atoms per cluster, which resulted in an energy of 2.5 eV/atom. The sputter area was set to 5 mm × 5 mm with a Zalar rotation.

The hematite photoanode was measured with a RENON MsAa-4 digital Mössbauer spectrometer using a ^57^Co in Rh matrix source for 14.4 keV γ-rays at room temperature. The spectra were registered in a 1024-channel analyzer and folded to 512 channels when analyzed.

## Results and Discussion

**Figure 1:**
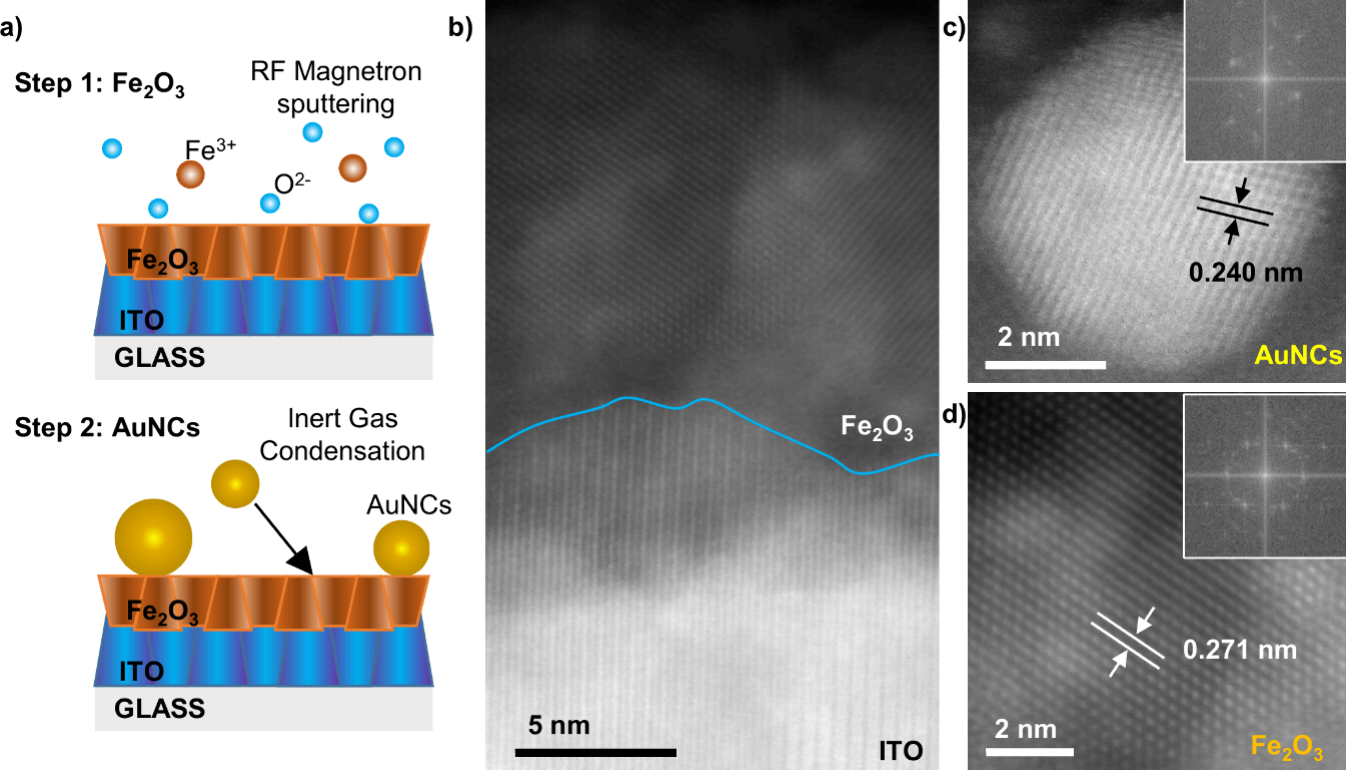
(a) Fabrication routes of hematite and AuNCs/α-Fe_2_O_3_ photoanodes using radio frequency reactive magnetron sputtering and inert gas condensation techniques, (b) HR-STEM image of the AuNCs/α-Fe_2_O_3_ thin film cross-section, (c) zoom-in Au nanograin with a measured *d*-spacing corresponding to the (111) plane, and (c) hematite grain with the measured *d*-spacing corresponding to the (2–11) plane (the insets show the corresponding FFT transformations).

Figure 1a presents the fabrication routes for thin films of hematite and AuNCs-decorated hematite (AuNCs/α-Fe_2_O_3_). The top row corresponds to the pristine α-Fe_2_O_3_ film, and the bottom row corresponds to the α-Fe_2_O_3_ film decorated with gold nanoclusters. The topography of the thin films studied by SEM and AFM is presented in the Supporting Information in Figure S1. In-depth morphological studies of thin film FIB cross sections were performed using STEM-HAADF techniques. The microstructure was resolved with atomic resolution (Figure 1b), revealing the polycrystalline structure of the thin films deposited onto the ITO substrate. The average size of the crystallite is approximated to 5 nm (see Figure 1c,d). The insets in Figure 1c,d show the FFT transformation of the HAADF-STEM images of AuNCs and hematite nanograin, confirming the crystallinity of AuNCs and the α-Fe_2_O_3_ phase, which corresponds to the hcp crystal structure.^20^ A detailed description of this analysis is provided in the Supporting Information in Figure S2. The measured and theoretical crystallographic parameters (interplanar distances and angles) corresponding to the α-Fe_2_O_3_ phase are summarized in Tables S1 and S2. The film thickness is equal to 10 nm, which is consistent with the value measured by the contact profilometer. The studies reported by Steier^21^ showed already that, in the case of atomic layer deposition (ALD) thin films, such a thickness provided the highest internal quantum efficiency.

**Figure 2:**
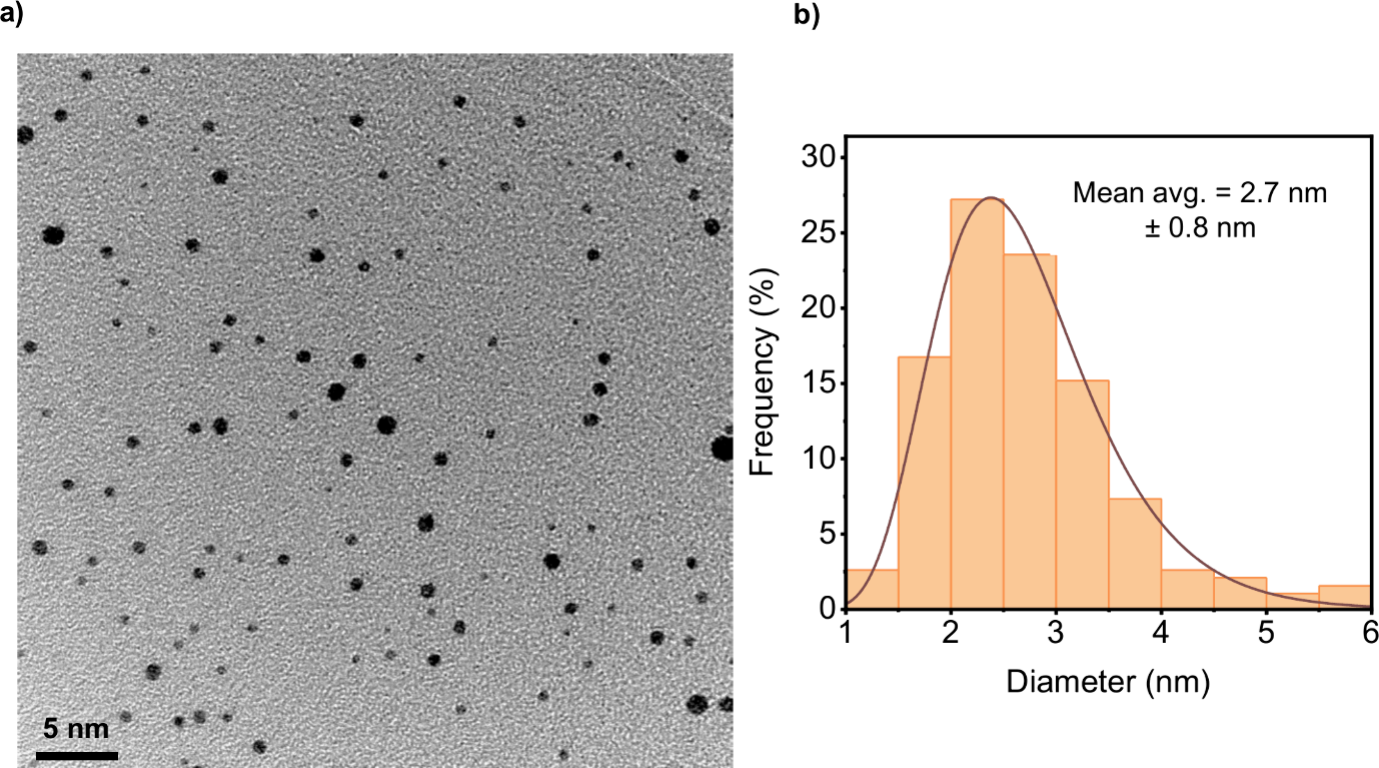
TEM analysis of the top layer of gold nanoclusters deposited using the inert gas condensation technique: (a) the distribution of nanoclusters and (b) a histogram.

Additional characterization of the size and distribution of Au nanograins sputtered in the same process was performed by using the TEM method. Their distribution can be seen in Figure 2a, and the corresponding histogram is shown in Figure 2b. The surface coverage of the Au layer is equal to 2.3%, and the mean size of the particle was equal to 2.7 ± 0.8 nm.

Several groups have already reported that even a slight modification of deposition conditions can have a great impact on the electrical properties of hematite, changing the room conductivity by 6 orders of magnitude.^22^ Therefore, to determine solely the role of AuNCs at the interface, it is important to ensure that the subsequent modification does not alter the intrinsic properties of the hematite layer.

**Figure 3:**
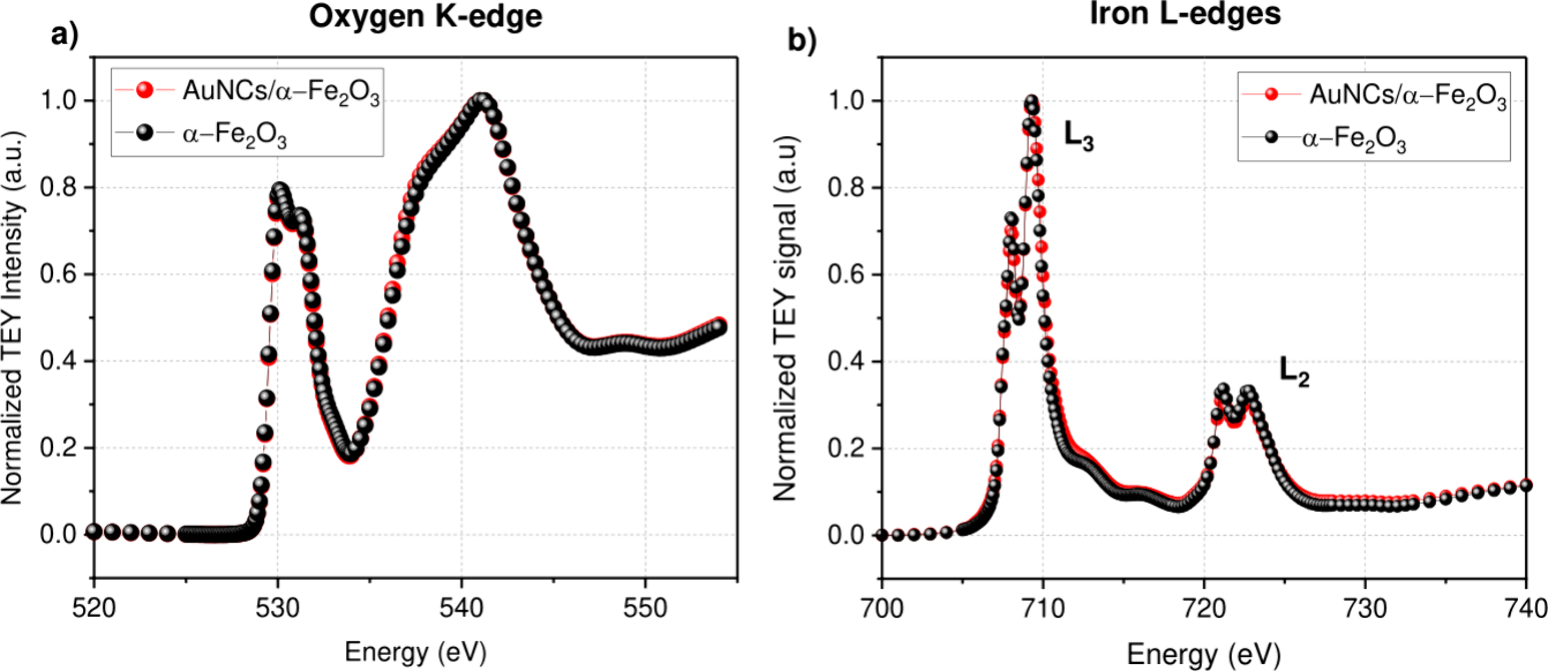
XAS of the thin film of hematite deposited on the ITO substrate: (a) oxygen K edge and (b) iron L_2,3_ edges.

The electronic structure of the material was investigated by using XAS. Figure 3 demonstrates the normalized spectra at the oxygen K edge (Figure 3a) and the iron L edge (Figure 3b) for a thin film of hematite. Iron L-edge spectra consist of two regions, corresponding to L_3_ and L_2_ resonances. Both resonances show characteristic splitting and modulations originating from the final state (2p^5^3d^6^) multiplet structure.^23^ The observed shape is typical of that reported for thin films and nanostructures of hematite.^24,25^ The oxygen K-edge spectra reveal a double peak pre-edge structure around 530 eV, resulting in the hybridization of oxygen 2p states with the crystal field split 3d orbitals of iron.^26,27^ The energy splitting depends on the iron–oxygen stoichiometry and crystal structure. The large splitting energy of more than 1.4 eV is characteristic of α-Fe_2_O_3_.^28^

The complementary chemical phase analysis was performed with conversion electron ^57^Fe Mössbauer spectroscopy. The hyperfine parameters—the isomer shift *I*_S_ = 0.37 mm/s, quadrupole shift *Q*_S_ = −21 mm/s, and hyperfine magnetic field *B*_Hf_ = 50.1 T—also depict the polycrystalline hematite phase. A detailed description of this analysis with the Mössbauer spectrum (see Figure S3) and the fit of hyperfine parameters (see Table S3) can be found in the Supporting Information.

The energy-dispersive X-ray spectroscopy (see Figure S4) revealed the same intensity of peaks corresponding to Fe and O, confirming the same elemental composition of α-Fe_2_O_3_. The detection limit does not allow for observation of the signal originating from Au nanoclusters.

**Figure 4:**
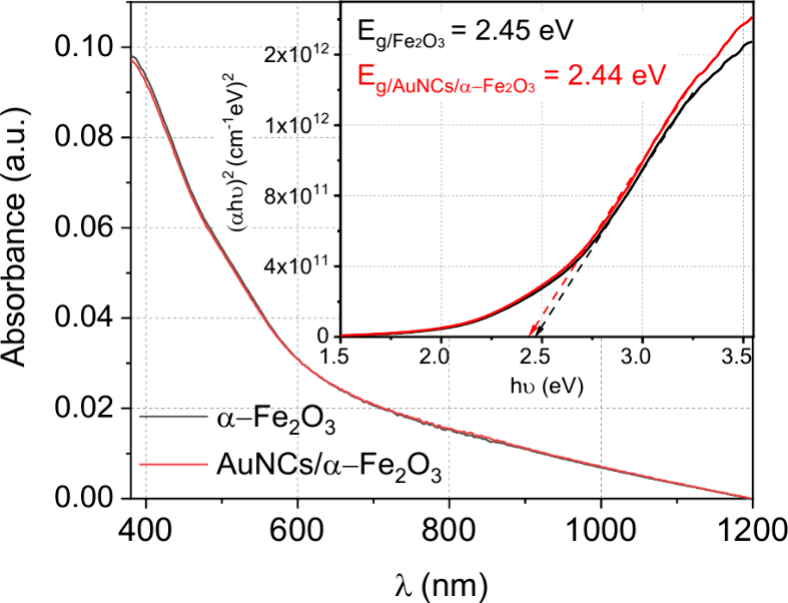
Optical absorption of hematite thin films obtained from UV–Vis measurements. The inset represents the Tauc plot for the indirect transition (γ = 2) and the determined optical band gaps equal to 2.45 and 2.44 eV.

The optical properties were investigated by using UV–Vis spectroscopy and are presented in Figure 4. The inset presents the Tauc plot for the indirect transition, assuming γ = 2 in the Tauc formula (eq 2).^29^
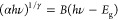
2

Extrapolating the linear part of the Kubelka–Munk function to (α*h*υ) = 0 allows us to estimate the band gap, which amounts to 2.45 and 2.44 eV, for α-Fe_2_O_3_ and AuNCs/α-Fe_2_O_3_. The value based on the intersection of the 2.45 eV was already observed for α-Fe_2_O_3_ nanoclusters with a crystal size of 4 nm or thin films with different crystallites deposited by DC magnetron sputtering.^22^ The blue shift of the energy band can be explained by the Brus model, which corrects the bulk value for the smaller size of the crystallites. In the absorption spectra, we do not observe plasmonic contribution, as expected for such a low density of Au nanoclusters; therefore, we can make a statement that the optical enhancement by Au nanoclusters is negligible in our studies.

**Figure 5:**
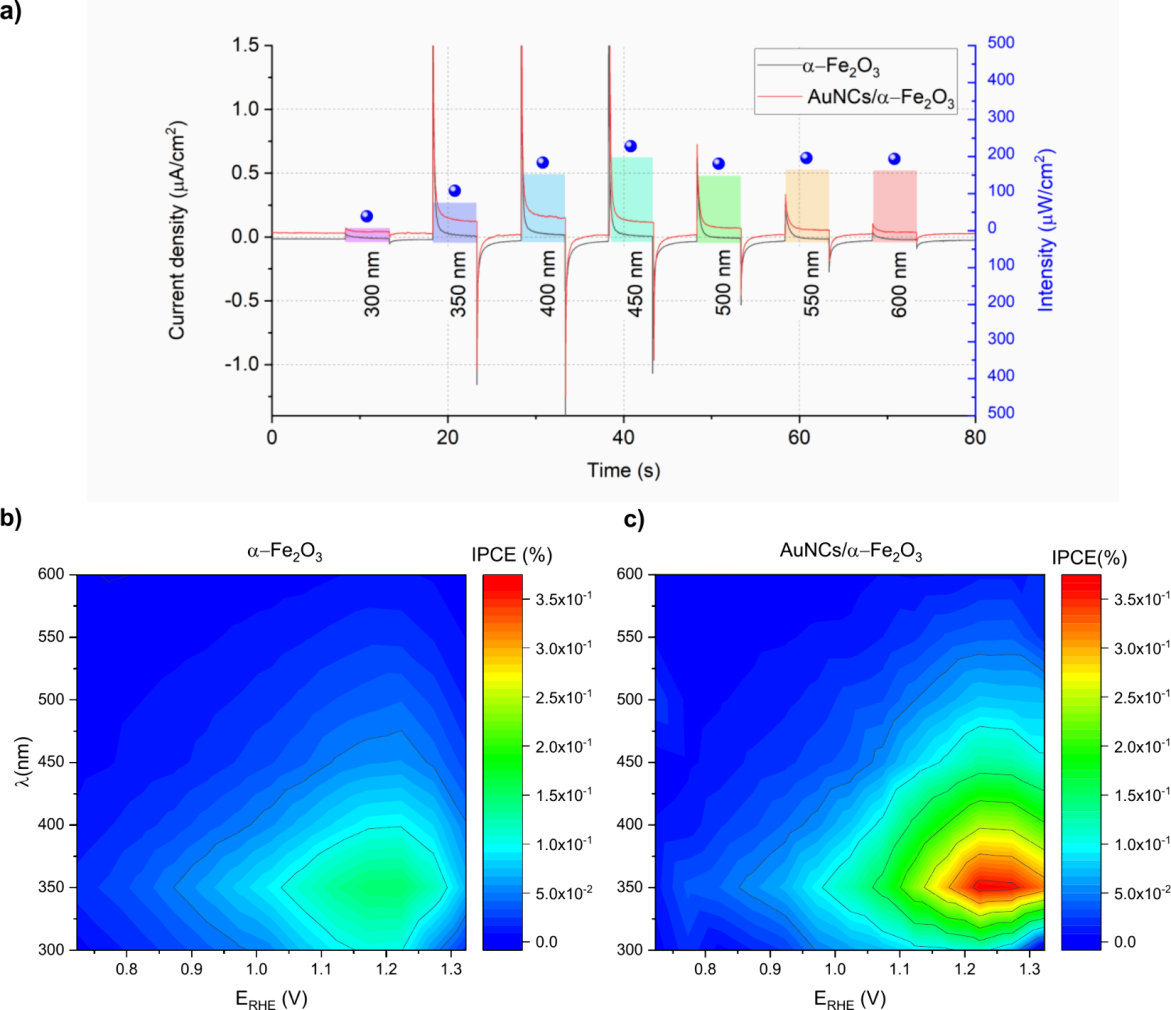
(a) Time evolution of photocurrents during exposure to light pulses with selected wavelengths recorded at *E*_RHE_ = 1.025 V. The height of the color bar corresponds to the light intensity. Photocurrent maps as a function of potential and wavelength for (b) pristine and (c) AuNC-decorated hematite.

Photocurrent measurements were performed by setting the electrode potential and pulsing the light with a particular wavelength. The potential electrode *E*_Ag/AgCl_ ranged from −300 to 300 mV (vs Ag/AgCl) with a step of 50 mV, and the wavelength λ ranged from 300 to 600 nm, with a step of 50 nm. The values were converted to *E*_RHE_ using (eq 1), described in the Experimental Section. The evolution of transient photocurrents during chopped light illumination at a selected value of applied potential (*E*_RHE_ = 1.025 V) is illustrated in Figure 5a. The colors reflect the intensity of the light, extracted using a monochromator from the white-spectrum 150 W xenon lamp. The red curve corresponding to AuNCs/α-Fe_2_O_3_ already indicates the photocurrent enhancement.

The photocurrent maps for the as-measured values (see Figure S5a,b) were recalculated to the incident photon-to-current efficiency (IPCE) according to eq 3:
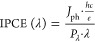
3where *J*_ph_ is the photocurrent density, *h* is Planck’s constant, *c* is the speed of light, *e* is the elementary charge, *P*_λ_ is the incident power density, and λ is the wavelength. The maps of IPCE values for both photoanodes are represented in Figure 5b,c. The IPCE enhancement, measured as the difference between the two films, is shown in Figure S5c. The light power and light intensity are plotted in Figure S5d.

The IPCE values, recorded at *E*_RHE_ = 1.025 V, are in the same range as the one reported for a similar system of the ALD α-Fe_2_O_3_ thin film with a thickness of 10 nm without a TiO_2_ underlayer.^21^ The drop in IPCE values for wavelengths lower than 300 nm could be an experimental artifact originating from the low light intensity. The AuNCs/α-Fe_2_O_3_ photoanode exhibits enhanced activity over a broader potential and spectral range. The IPCE peak shifted to more electropositive potential values. These changes are not attributed to shifts in the band positions but rather to differences in charge carrier lifetime and a significantly reduced rate of charge carrier recombination. Additionally, the IPCE values in the region corresponding to the 350 nm peak are twice as high as those observed for α-Fe_2_O_3_.

We have also investigated the relative change between the maximum of the photocurrent peak and the steady-state photocurrent. The results presented in Supporting Information (Figure S6a–c) show the changes in the photocurrent peak maximum and the steady-state photocurrent (Δ*J*_max–ss_) for α-Fe_2_O_3_ and AuNCs/α-Fe_2_O_3_ at different potentials and wavelengths. The photocurrent peak reflects charge accumulation at the surface, which starts to occur at 0.8 V, as observed in the work by Le Formal et al.^30^ The reduction in charge accumulation for AuNCs/α-Fe_2_O_3_ compared to the pristine film indicates that AuNCs attenuate charge buildup at the surface, which correlates well with the observed enhancement in IPCE values (Figure S5c). This finding is in agreement with previous studies on hematite films coated with a cobalt catalyst,^30^ where a similar effect was observed. Our results further support the hypothesis that modifying the photoanode surface can influence charge dynamics and suggests that AuNCs enhance hole injection into the electrolyte, potentially improving the overall photoelectrochemical performance.

**Figure 6:**
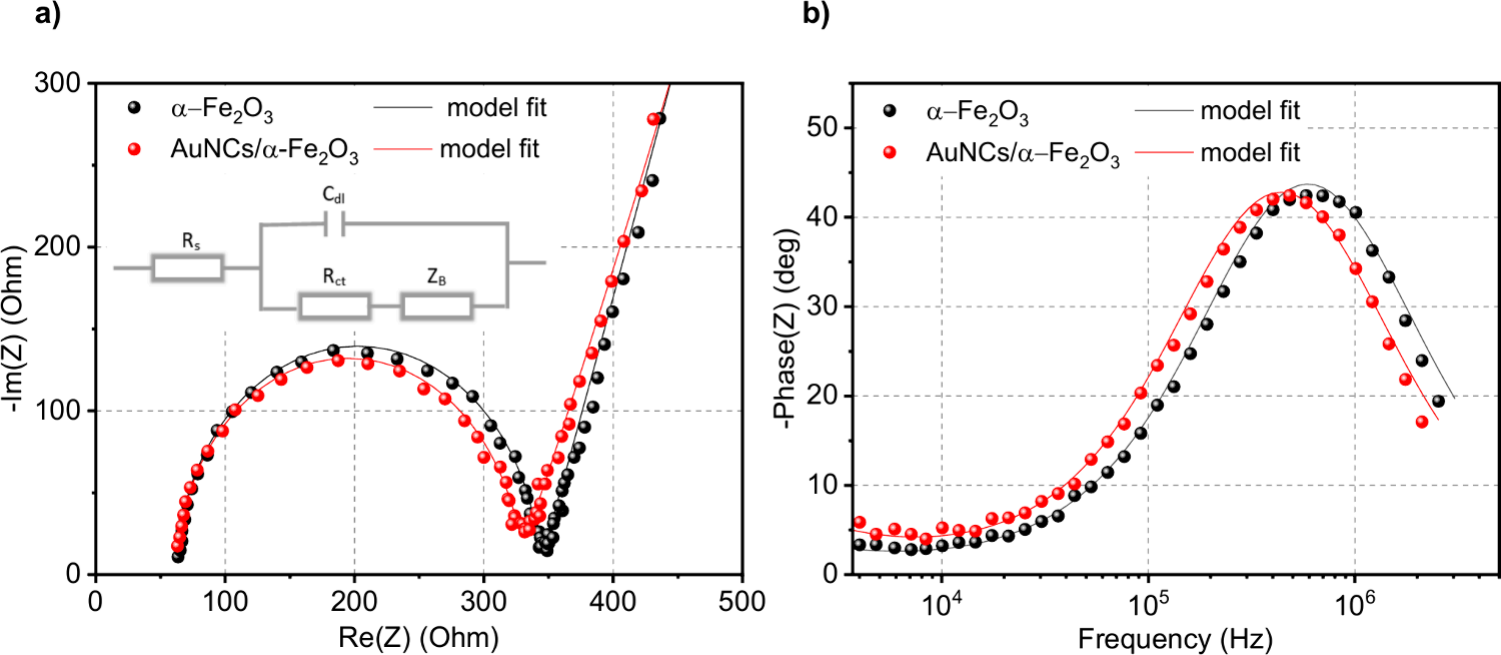
(a) Nyquist and (b) Bode plots recorded for α-Fe_2_O_3_ and AuNCs/α-Fe_2_O_3_ electrodes.

The EIS measurements were performed to evaluate the dynamics of charge transfer. In previous articles, the EIS properties of hematite were investigated in detail using many different models.^31−37^ The EIS spectra obtained in current studies, visible in Figure 6a, were analyzed using an equivalent circuit composed of *R*_s_ (contact series resistance), *R*_ct_ (resistance of charge carriers from surface states to the electrolyte), and *C*_dl_ (double layer capacitance). In the recorded spectra, an angle characteristic for the low-frequency diffusive part significantly exceeds −π/4; therefore, the fitting Bisquert element (*Z*_B_) describing the anomalous linear diffusion was utilized. The impedance of the element is described in the work by Bisquert and Compte^38^ with eq 4:
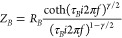
4where γ ≤ 1, τ_B_ is the diffusion time constant, *R*_B_ is the resistance associated with the diffusion mechanism, *f* is the frequency in Hz, and *i* is the imaginary number (*i*^2^ = −1).

The semicircle radius corresponding to charge transfer at the solid-state-electrolyte interface is lower in the case of AuNCs/α-Fe_2_O_3_, indicating a *R*_ct_ amounting to 264.3 Ohm (see Figure 6a). It can be concluded that modification of α-Fe_2_O_3_ with AuNCs results in an acceleration of the charge transfer kinetics. This, in turn, may limit the effect of recombination of photoexcited electrons and holes and thus can result in improvement of photocurrent activity through enhancement of charge carrier mobility, as was demonstrated by differences in photocurrent intensities in Figure 5a,b.

The Bode plot in Figure 6b indicated that modification of α-Fe_2_O_3_ with AuNCs also resulted in a shift of the characteristic maximum frequency peak toward the lower-frequency range. The electron lifetime (τ_n_) calculated based on the observed peak frequency (*f*_max_) corresponding to charge transfer at the material/electrolyte interface using eq 5(39) amounted to 0.27 and 0.36 μs for α-Fe_2_O_3_ and AuNCs/α-Fe_2_O_3_, respectively (Table 1).
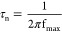
5

**Table 1:** Summary of the Parameters Determined Based on the Recorded EIS Spectra

	*R*_s_	*C*_dl_	*R*_ct_	*R*_B_	τ_B_	γ_B_	*f*_max_	τ_n_
	Ohm	F	Ohm	kOhm	s		Hz	μs
Fe_2_O_3_	64.39	2.225 × 10^–9^	276.7	2.50	0.036	0.50	581,776	0.27
	64.81	1.944 × 10^–9^	277.1	2.53	0.023	0.50	637,266	0.25
	64.67	2.507 × 10^–9^	275.5	2.46	0.032	0.49	637,266	0.25
average values	64.62	2.22 × 10^–9^	276.4	2.50	0.030	0.050	618,769	0.26
Std. Dev.	0.21	0.282 × 10^–9^	0.833	0.04	0.007	0.006	32,037	0.01
AuNCs/Fe_2_O_3_	62.59	3.113 × 10^–9^	263.7	9.29	0.094	0.49	440,766	0.36
	61.06	3.128 × 10^–9^	264.6	9.15	0.086	0.49	440,766	0.36
	61.14	3.193 × 10^–9^	264.6	9.06	0.075	0.49	483,317	0.34
Average values	61.60	3.145 × 10^–9^	264.3	9.17	0.085	0.49	454,950	0.35
Std. Dev	0.86	0.043 × 10^–9^	0.520	0.12	0.009	0	24,567	0.01

The Bode plot indicated that the presence of AuNCs successfully limited charge carrier recombination, which in turn led to a significant extension of the electron lifetime compared to pure α-Fe_2_O_3_.

**Figure 7:**
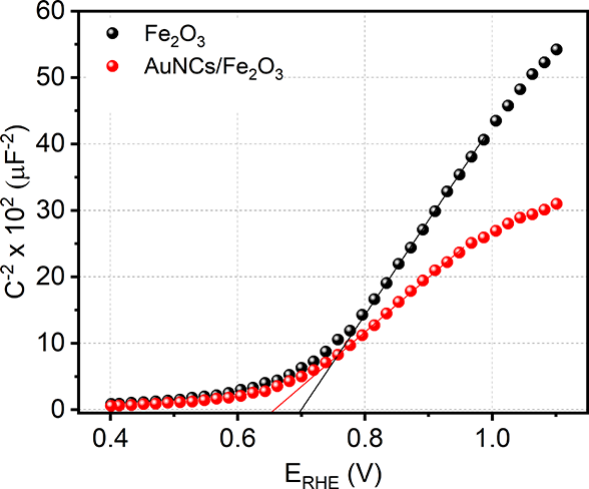
Mott–Schottky plots of α-Fe_2_O_3_ and AuNCs/α-Fe_2_O_3_ recorded at 1 kHz.

Mott–Schottky analysis was performed in order to analyze the charge carrier density for particular materials resulting from the formation of the Schottky barrier at the material/electrolyte interface and is presented in Figure 7. The Mott–Schottky approximation of the capacitance–voltage dependency is given by eq 6:
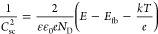
6where ε is the dielectric constant of the semiconductor, ε_0_ is the vacuum permittivity (8.85 × 10^–12^ F/m), *e* is the elementary charge (1.602 × 10^–19^ C), *N*_D_ is the concentration of electron donors, *E* is the applied potential, *E*_fb_ is the flat band potential, *k* is the Boltzmann constant (1.381 × 10^–23^ J/K), and *T* is the temperature. The knowledge of the imaginary part of impedance (*Z*_im_) at a given frequency enables the determination of the capacitance of the space charge region (*C*_sc_) expressed by eq 7:
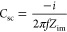
7

The positive slope of the Mott–Schottky dependence indicated the n-type conductivity of the synthesized materials. Electron donor density and flat band potential (*E*_fb_) can also be determined based on the slope of the Mott–Schottky plot and the extrapolation of the dependence to *C*_sc_ = 0.

The determined values of *E*_fb_ are equal to 0.65 and 0.7 V (vs RHE) for α-Fe_2_O_3_ and AuNCs/α-Fe_2_O_3_, respectively. The very slight shift in the localization of the flat band potential toward a less positive direction, resulting from the presence of AuNCs, may be attributed to differences in surface state density and variations in double-layer capacitance (M–S plot). The AFM results indicate that the presence of AuNCs resulted in a slight decrease in surface roughness. For α-Fe_2_O_3_, the average roughness (Ra) was 1.46, while for AuNCs/α-Fe_2_O_3_, the Ra parameter was 1.14, suggesting a higher electrochemically active surface area (EASA) for α-Fe_2_O_3_. EASA is a crucial factor that determines the conditions for charge accumulation. In 1 M NaOH (a concentrated electrolyte), the higher capacitance of α-Fe_2_O_3_ may effectively shield the charge at the semiconductor surface. This, in turn, could limit the band bending effect and shift the flat band potential toward a more electropositive range. On the other hand, the AuNCs, because of the different work function value (ca. 5.15 eV),^40^ compared to sputtered films of pristine α-Fe_2_O_3_ (5.4 −5.7),^41^ may also act as electron donors. This in turn results in the shifting of the α-Fe_2_O_3_ Fermi level and *E*_fb_ toward the electronegative direction. The mutual localization of *E*_fb_ for α-Fe_2_O_3_ and AuNCs/α-Fe_2_O_3_ results mainly from the mutual interplay of these factors.

The slope of the Mott–Schottky dependence expressed by  allows the determination of the charge carrier density for the obtained materials. Comparing the slopes of Mott–Schottky dependences, it may be observed that charge carrier density is even 1.7 times higher for AuNCs/α-Fe_2_O_3_ than for pure hematite. It is in good agreement with the data obtained from Nyquist and Bode plots and shows that AuNCs affect the charge carrier density through the limitation of the recombination and thus extension of electron lifetime. A higher charge carrier density may be considered to be a crucial factor responsible for the enhancement of the intensity of generated photocurrents.

It should be noted that the observed shift of the *E*_fb_ value toward a more electronegative direction and the changes in the Mott–Schottky slope resulting from the presence of Au nanoclusters are in good agreement with the data reported by Li for the AuNCs/α-Fe_2_O_3_-nanocomposite material.^42^

**Figure 8:**
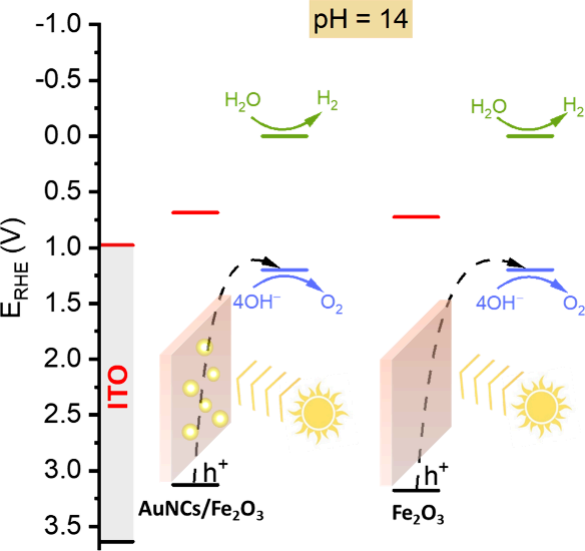
Charge transfer diagram for AuNCs/α-Fe_2_O_3_ and α-Fe_2_O_3_ thin films.

The band localization for both materials is very similar, as can be seen on the energy diagram shown in Figure 8. In both cases, the holes take place in the oxidation of hydroxyl anions (4OH^–^) to produce oxygen, and the photoexcited electrons are involved in the hydrogen reduction reaction occurring on the surface of Pt.

The Mott–Schottky analysis was conducted with an awareness of the limitations associated with using a single-frequency method. This approach was chosen to ensure a direct comparison between the two materials under identical conditions. By measuring both photoanodes at the same frequency, we were able to analyze the variations in capacitance resulting from the presence of AuNPs.

Furthermore, the relative positioning of the valence and conduction band edges with respect to the redox potentials of water reduction and hydroxyl oxidation reactions suggests that even significant differences arising from the use of alternative frequencies or methodologies—such as step polarization of the electrode with EIS spectrum analysis—would not alter the fundamental mechanism of the photoelectrocatalytic processes.

## Conclusions

Our study demonstrates that the decoration of hematite thin films with Au nanoclusters significantly enhances photoelectrochemical performance by improving charge transfer kinetics. Keeping a low surface coverage of 2%, we eliminated the plasmonic contribution and achieved a 2-fold increase in photoelectrochemical activity. This enhancement can be elucidated by EIS and is attributed to increased charge carrier density, extended carrier lifetime, and attenuated charge carrier accumulation at the surface. These findings offer valuable insights into the development of more efficient photoelectrodes for solar-driven water-splitting applications.

## Supporting Information Available

SEM, AFM, analysis of SAED, conversion electron Fe^57^ Mössbauer spectroscopy, EDX, photocurrent spectroscopy, and UPS results (PDF)
