# Supplementary material for: Enhanced Charge Transfer Kinetics in Hematite Photoanodes via Au Nanoclusters—Toward Efficient Light-Driven Hydrogen Generation
Source: Langmuir. 2025 May 1;41(18):11349–57. doi: 10.1021/acs.langmuir.4c04843 (PMC12080339; doi:10.1021/acs.langmuir.4c04843)
Supplement: Supplementary file 1 — la4c04843_si_001.pdf [file la4c04843_si_001.pdf]

Supporting Information

Enhanced charge transfer kinetics in hematite photoanodes via Au nanoclusters – toward efficient light-driven hydrogen generation

Aleksandra Szkudlarek[*1], Kamila Kollbek[1], Krzysztof Mech[1], Krzysztof Maćkosz[2,3], Mateusz Marzec[1], Vitaliy Bilovol[1] and Marcin Sikora[1,4]*

[1]AGH University of Krakow, Academic Centre for Materials and Nanotechnology, av. Mickiewicza 30, 30-059 Krakow, Poland

[2]AGH University of Krakow, Faculty of Physics and Applied Computer Science, Department of Solid State Physics, av. Mickiewicza 30, 30-059 Krakow, Poland

[3]EMPA, Swiss Federal Laboratories for Material Science and Technology, Advanced Materials and Surfaces, Mechanics of Materials and Nanostructures, Feuerwerkerstrasse 39, 3602 Thun, Switzerland

[4]National Synchrotron Radiation Centre SOLARIS, Jagiellonian University, Czerwone Maki 98, 30-392, Krakow, Poland

Scanning Electron Microscopy and Atomic Force Microscopy

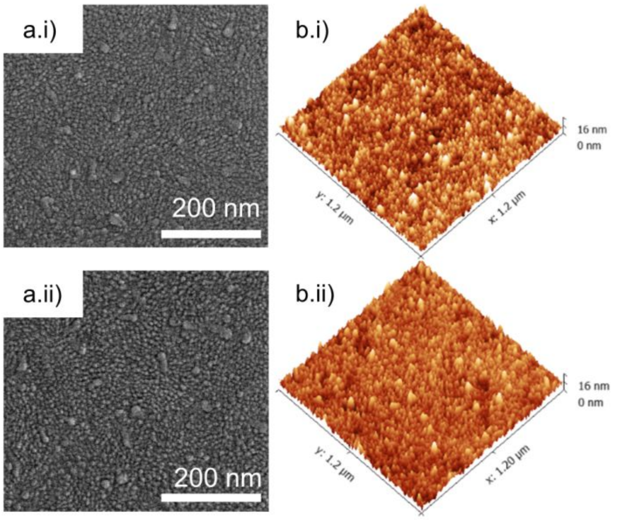

Figure S1. a) SEM images, b) AFM scans of hematite photoanodes (i) pristine α-Fe_2O_3 (ii) AuNCs/α-Fe_2O_3 deposited on ITO glass substrates.

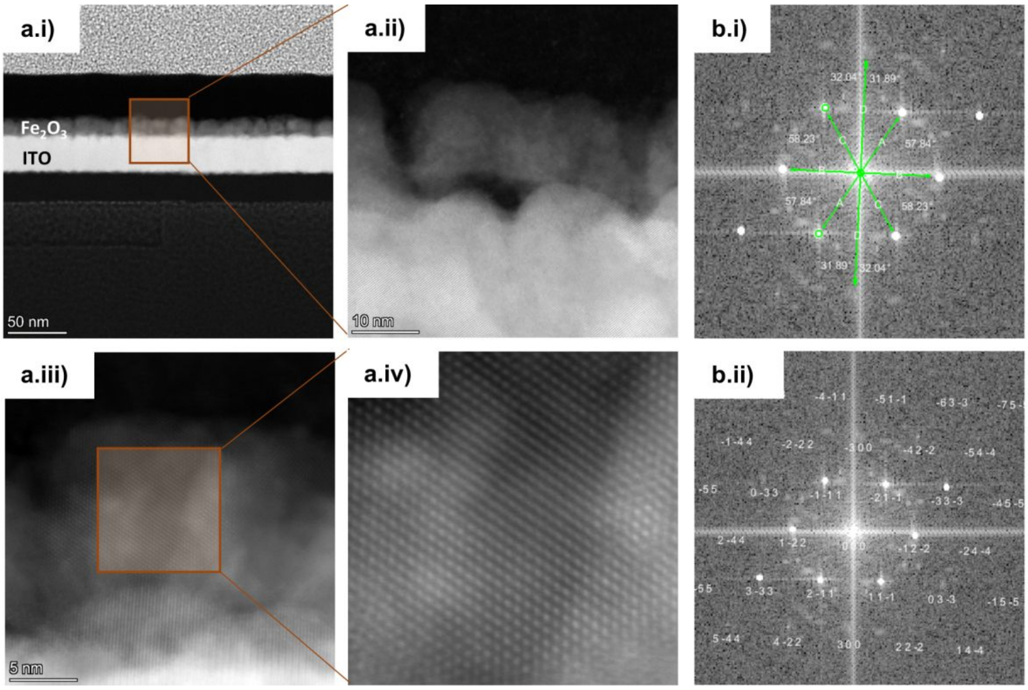

Figure S2. a)(i-iv) STEM- HAADF analysis of α-Fe_2O_3 nanograin: i) cross section of α-Fe_2O_3 at ITO thin film b) i) Fast Fourier Transform of (a.iv) image and with the values of measured interplanar angles ii) crystallographic indices were adjusted with CrystBox software [1].

Table S1. Measured values of the d-spacing obtained from FTT transformation of Fig.S2a.iv STEM-HAADF image, compared to the d-spacing values calculated from hematite .cif file [2].

Vector	Plane	d-spacing (measured)		d-spacing (theoretical)		difference	
		[nm]	[1/nm]	[nm]	[1/nm]	[nm]	[1/nm]
A	2 – 1 1	0.2714	3.6846	0.2472	4.045	0.0242	-0.3604
B	1 -2 2	0.2476	4.03877	0.236	4.237	0.0116	-0.1982
C	-1- 1 1	0.2747	3.64033	0.2472	4.045	0.0275	-0.4047
D	-3 0 0	0.1504	6.64894	0.1451	6.893	0.0053	-0.2441

Table S2. Measured values of the interplanar angles obtained from hematite grain (shown in Fig. S2a.iv STEM-HAADF image), compared to the theoretical values calculated from hematite .cif file.

Vectors	Angles (measured)	Angles (theoretical)	Difference
A,B	57.84	58.42	-0.58
B,C	58.23	58.42	-0.19
C,D	32.04	31.58	0.46

D,A	31.89	31.58	0.31

Conversion Electron ^{57}Fe Mössbauer spectroscopy

The ^{57}Fe Mössbauer spectroscopy technique is a suitable one to distinguish and identify different phases of iron oxides and eventual impurities. The set of hyperfine parameters (isomer shift I_S, quadrupole shift Q_S and hyperfine magnetic field B_{Hf}) is characteristic for a given iron oxide phase. Typically, a spectrum of well-crystallized hematite will show a sextuplet with sharp lines reflecting the presence of Fe^{3+} ions only. The fitted hyperfine parameters of our sample and the reference data from the literature are summarized in Table S3. The value of a hyperfine magnetic field of about 50 T for hematite is significantly higher than the 48 T, expected for magnetite [4]. Also, the isomer shift, which reflects the valence state of Fe^{3+}, is close to the value of 0.36 mm/s (referred to α-Fe), typically observed for hematite. Eventually, the value of quadrupole shift is the strongest argument confirming that the sample is grown with a corundum structure (for the cubic maghemite in the spinel phase, the quadrupole shift is equal to zero [5,6]). The intensity of the spectral lines is associated with an orientation of the local magnetization at the nuclei with respect to the γ-beam [7,8] and it is proportional to the product of the transition probability and the angular factors ($F(\theta)=2\sin^2\theta$ for θ, $\Delta m=0$, $F(\theta)=1+\cos^2\theta$ for $\Delta m=\pm1$). For a polycrystalline isotropic sample, the intensity ratio of the six lines follows the pattern 3:2:1:1:2:3. In this case, the average angle is expected to be about 54°. The deviation from this value is due to the preferential orientation of magnetic moments in thin films due to the small thickness of the films. In the case of our thin film, this angle was close to 70°, which implies some degree of magnetic texture in the hematite film.

Comparing the spectra to the results of the RF sputtered thin films, reported by Uribe et al. [9], Kment et al. [3], similar values of hyperfine magnetic field and isomer shift were obtained. The decreasing value of quadrupole splitting can be associated with reducing the size of the crystallites; this effect was observed during the milling process of commercial powder [10]. From the Mössbauer analysis, we can draw a conclusion that our film is composed of small nanocrystallites of hematite.

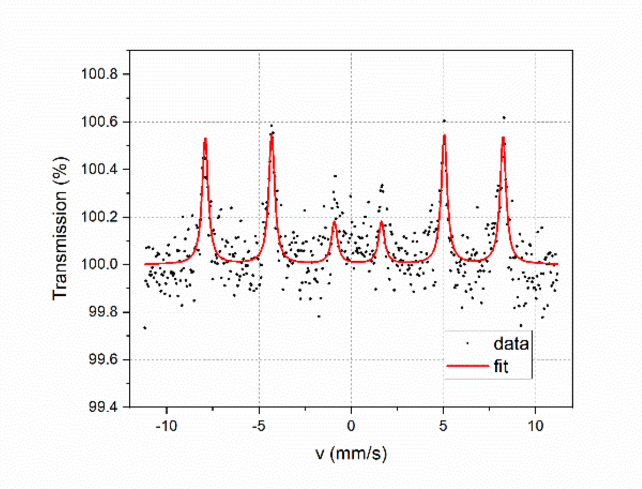

Figure S3. Conversion electron [57]Fe Mössbauer spectra (CEMS) for thin film of hematite.

Energy Dispersive Spectroscopy

Table S3. Hyperfine parameters for selected forms of hematite.

Material	Hyperfine magnetic field (T)	Isomer shift (mm/s)	Quadrupole splitting (mm/s)	Ref.
Polycrystalline commercial hematite powder – unmilled	50.7	0.36	-0.20	[10]
Fe_2O_3 deposited by RF on Fe foil (thickness of	50.6	0.27	-0.14	[9]
Fe_2O_3 deposited by pulsed magnetron sputtering	50.8	0.32	–0.18	[3]
Fe_2O_3 deposited by RF on ITO (thickness of 10 nm)	50.1	0.37	-0.21	This work

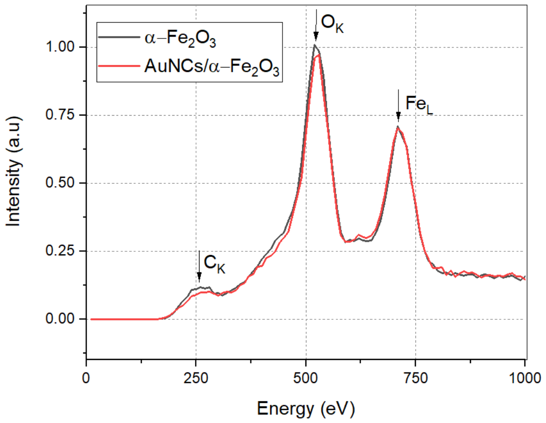

Figure S4. Intensities of Energy Dispersive X-ray peaks corresponding to the elemental composition of α-Fe_2O_3 and AuNCs/α-Fe_2O_3 thin films.

Photorcurrents Spectroscopy

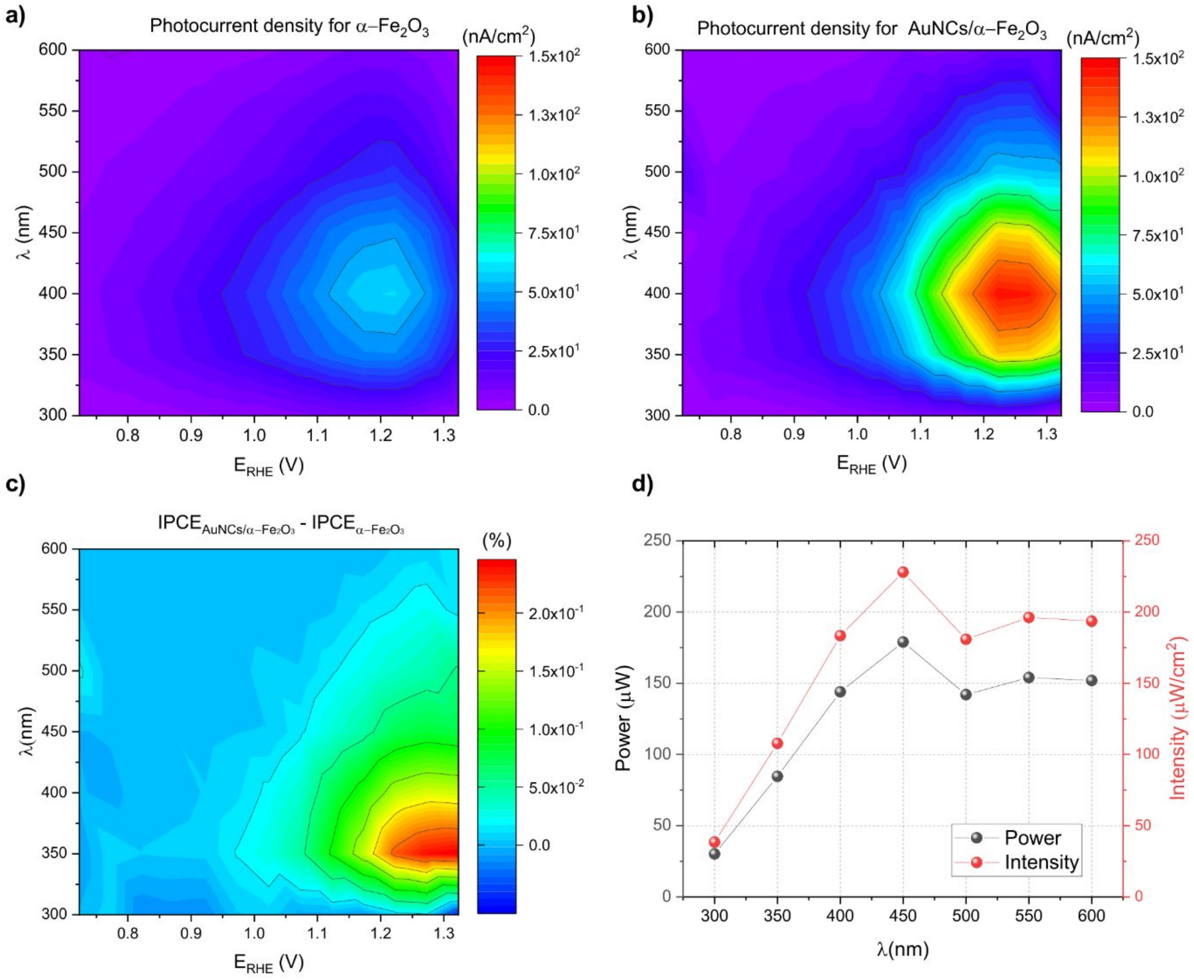

Figure S5. As measured photocurrent values for a) α-Fe_2O_3 and b) AuNCs/α-Fe_2O_3 photoanode and c) difference in IPCE values d) photodiode power converted to light intensity, used for determining IPCE value.

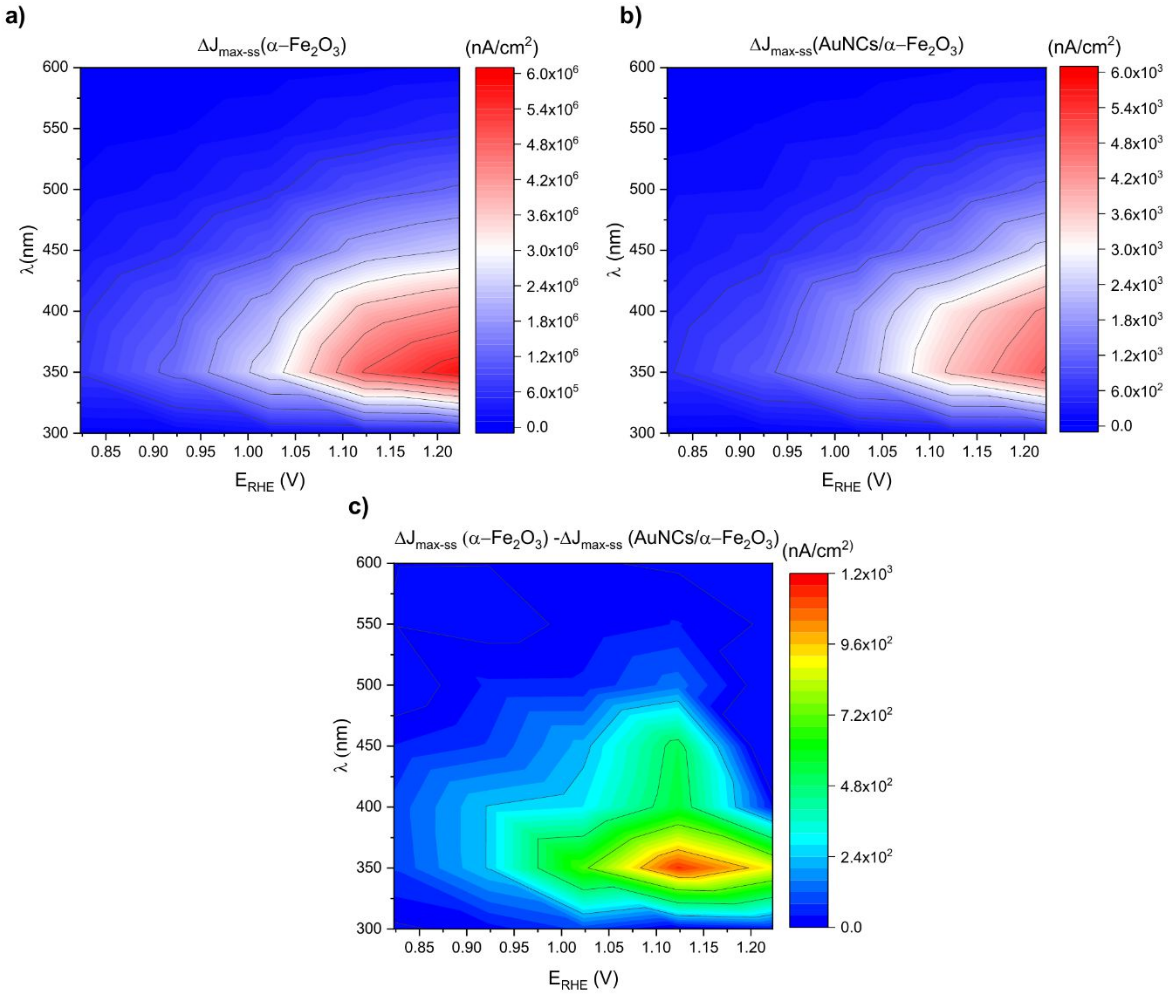

Figure S6. Difference between the maximum transient and steady-state photocurrents ($\Delta J_{max\text{-}ss}$) measured at potentials ranging from 0.823 to 1.223 V and light wavelengths from 300 nm to 600 nm for (a) α-Fe_2O_3, (b) AuNCs/α-Fe_2O_3, and (c) the difference in values observed between the two materials.

Ultraviolet photoelectron spectroscopy (UPS)

The values of the measured work function using the UPS technique, although not measured in UHV conditions were equal to 4.41 eV and 4.47 eV for the α-Fe_2O_3 and AuNCs/α-Fe_2O_3 thin films, before sputtering of Ar clusters and 4.36 eV after the sputtering process. These values are much lower than expected for hematite thin films (approximately 5.4 – 5.8 eV), but they correspond quite well to reported values 4.4-4.5 eV of ITO [11]. The ultraviolet photoelectron spectroscopy (UPS) measurements were performed in a PHI VersaProbeII apparatus (ULVAC-PHI, Chigasaki, Japan) using He I line (21.22 eV) from a UHV gas discharge lamp. The acceleration potential of − 5 V was applied to the sample leading to a much more pronounced secondary electron cut off (SE cut-off). The work function (measured as the difference between photon energy and SE cut-off position) and the hole injection barrier (given by

the difference of the substrate Fermi level and the HOMO onset of the material) were determined. For each UPS spectrum the emission features originating from the secondary line excitations of the He I gas discharge were subtracted. The measurement times were kept as short as possible to avoid any possible degradation of the examined materials during the exposure to UV radiation. The measurements have been done before and after Argon Gas Cluster Ion Beam sputtering of samples. The sputtering process was carried out with approximately 4000 atoms per cluster which resulted in energy of 2.5 eV/atom. Sputter area was set to 5 x 5 mm^2 with Zalar rotation.

References

1. Klinger, M. More features, more tools, more *CrysTBox*. *J. Appl. Crystallogr.* **50**, 1226–1234 (2017).
2. Crystallography Open Database. *Information card for entry 9015964* http://www.crystallography.net/cod/9015964.html (2024).
3. Kment, S. *et al.* Photoanodes with Fully Controllable Texture: The Enhanced Water Splitting Efficiency of Thin Hematite Films Exhibiting Solely (110) Crystal Orientation. *ACS Nano* **9**, 7113–7123 (2015).
4. Freindl, K., Wojas, J., Kwiatek, N., Korecki, J. & Spiridis, N. Reversible oxidation–reduction of epitaxial iron oxide films on Pt(111): Magnetite–hematite interconversion. *J. Chem. Phys.* **152**, 054701 (2020).
5. Fock, J. *et al.* On the 'centre of gravity' method for measuring the composition of magnetite/maghemite mixtures, or the stoichiometry of magnetite-maghemite solid solutions, via ^{57} Fe Mössbauer spectroscopy. *J. Phys. Appl. Phys.* **50**, 265005 (2017).
6. Zakharova, I. N., Shipilin, M. A., Alekseev, V. P. & Shipilin, A. M. Mössbauer study of maghemite nanoparticles. *Tech. Phys. Lett.* **38**, 55–58 (2012).
7. Frauenfelder, H., Nagle, D. E., Taylor, R. D., Cochran, D. R. F. & Visscher, W. M. Elliptical Polarization of Fe 57 Gamma Rays. *Phys. Rev.* **126**, 1065–1075 (1962).
8. Kreber, E. & Gonser, U. Evaluation of relative line intensities in Mössbauer spectroscopy. *Nucl. Instrum. Methods* **121**, 17–23 (1974).

9. Uribe, J. D. *et al.* Hematite thin films: growth and characterization. *Hyperfine Interact.* **169**, 1355–1362 (2007).

10. André-Filho, J., León-Félix, L., Coaquira, J. A. H., Garg, V. K. & Oliveira, A. C. Size dependence of the magnetic and hyperfine properties of nanostructured hematite (α-Fe 2 O 3) powders prepared by the ball milling technique. *Hyperfine Interact.* **224**, 189–196 (2014).

11. Park, Y., Choong, V., Gao, Y., Hsieh, B. R. & Tang, C. W. Work function of indium tin oxide transparent conductor measured by photoelectron spectroscopy. *Appl. Phys. Lett.* **68**, 2699–2701 (1996).
